# The REFER (REFer for EchocaRdiogram) protocol: a prospective validation of a clinical decision rule, NT-proBNP, or their combination, in the diagnosis of heart failure in primary care. Rationale and design

**DOI:** 10.1186/1471-2261-12-97

**Published:** 2012-10-30

**Authors:** Lynda Tait, Andrea K Roalfe, Jonathan Mant, Martin R Cowie, Jonathan J Deeks, Rachel Iles, Pelham M Barton, Clare J Taylor, Marites Derit, FD Richard Hobbs

**Affiliations:** 1 Primary Care Clinical Sciences, Primary Care Clinical Sciences Building, University of Birmingham, Edgbaston, Birmingham, B15 2TT, UK; 2 General Practice & Primary Care Research Unit, University of Cambridge, Forvie Site, Robinson Way, Cambridge, CB2 OSR, UK; 3 Public Health, Epidemiology and Biostatistics, Public Health Building, University of Birmingham, Edgbaston, Birmingham, B15 2TT, UK; 4 Imperial College London (Royal Brompton Hospital), London, SW3 6LY, UK; 5 Health Economics Unit, University of Birmingham, Edgbaston, Birmingham, B15 2TT, UK; 6 Department of General Practice, University of Oxford, 23-38 Hythe Bridge Street, Oxford, OX1 2ET, UK

**Keywords:** Heart failure, Clinical decision rule, Diagnosis, Echocardiogram, NT-proBNP

## Abstract

**Background:**

Heart failure is a major cause of mortality and morbidity. As mortality rates are high, it is important that patients seen by general practitioners with symptoms suggestive of heart failure are identified quickly and treated appropriately. Identifying patients with heart failure or deciding which patients need further tests is a challenge. All patients with suspected heart failure should be diagnosed using objective tests such as echocardiography, but it is expensive, often delayed, and limited by the significant skill shortage of trained echocardiographers. Alternative approaches for diagnosing heart failure are currently limited. Clinical decision tools that combine clinical signs, symptoms or patient characteristics are designed to be used to support clinical decision-making and validated according to strict methodological procedures. The REFER Study aims to determine the accuracy and cost-effectiveness of our previously derived novel, simple clinical decision rule, a natriuretic peptide assay, or their combination, in the triage for referral for echocardiography of symptomatic adult patients who present in general practice with symptoms suggestive of heart failure.

**Methods/design:**

This is a prospective, Phase II observational, diagnostic validation study of a clinical decision rule, natriuretic peptides or their combination, for diagnosing heart failure in primary care. Consecutive adult primary care patients 55 years of age or over presenting to their general practitioner with a chief complaint of recent new onset shortness of breath, lethargy or peripheral ankle oedema of over 48 hours duration, with no obvious recurrent, acute or self-limiting cause will be enrolled. Our reference standard is based upon a three step expert specialist consensus using echocardiography and clinical variables and tests.

**Discussion:**

Our clinical decision rule offers a potential solution to the diagnostic challenge of providing a timely and accurate diagnosis of heart failure in primary care. Study results will provide an evidence-base from which to develop heart failure care pathway recommendations and may be useful in standardising care. If demonstrated to be effective, the clinical decision rule will be of interest to researchers, policy makers and general practitioners worldwide.

**Trial registration:**

ISRCTN17635379

## Background

Heart failure (HF) is a life-threatening, costly condition [1]. It affects at least 2.3% of adults over 45, rising to 4% in over 75 year olds [2]. HF markedly reduces quality and length of life [3], and treatment costs are high, second only to stroke and mainly due to high admission rates [4]; estimated to consume almost 2% (£751 million) of total NHS expenditure [5]. HF is a diagnostic challenge, as symptoms are non-specific and physical signs can be subtle [6-9]. Because outcomes in HF are linked to stage of disease and evidence-based treatments alter natural history as well as improve symptoms and prognosis [10-12], accurate early diagnosis and treatment is essential to reduce morbidity and mortality. As most patients with suspected HF are seen initially by GPs [6,13], the need for early and accurate diagnosis in primary care is essential to ensure optimum management and appropriate treatment is initiated rapidly.

Specialist review of symptoms and signs plus objective investigations, including echocardiography (Echo), is the established ‘gold standard’ for diagnosing left ventricular systolic dysfunction (LVSD) and increasingly suspected HF with a preserved ejection fraction (HFpEF) [14]. Diagnosing HF requires objective estimation of cardiac function (i.e. Echo) since determining the aetiology and stage of HF leads to different management choices such as initiation of angiotensin-converting enzyme (ACE) inhibitors [10], ß-blockers [11] and aldosterone antagonists in most patients with LVSD [15], cardiac resynchronization therapy for those with LVSD and broad QRS complex [1], or surgery where significant valve disease exists. These therapies improve symptoms, prognosis and quality of life, and can reduce healthcare utilisation and NHS costs. However, a difficulty is that performing Echo on all suspected HF patients would be costly as many patients are found not to have HF.

Diagnostic strategies can vary between GPs if a case of HF is suspected, but the most appropriate strategy is unclear. These include an initial clinical assessment of patient signs and symptoms using physical examination, and investigations such as lab blood tests or chest x-ray. Additionally, screening tests, such as electrocardiogram (ECG) and natriuretic peptide (NP) tests, where available, have been recommended by NICE as potential ‘rule out’ tests for HF to limit unnecessary referrals to echocardiography [16,17]. Routine clinical assessment takes place over multiple consultations, due mainly to diagnostic uncertainty and delays that occur in the referral pathway.

Diagnostic uncertainty in clinical practice, difficulties diagnosing HF and local organisational factors such as limited availability of diagnostic services, or delays inherent in the current referral system, create barriers to the early and accurate diagnosis of HF. Access to Echo is variable, often delayed, and limited by the significant skill shortage of trained Echocardiographers [14,16,18,19]. As a consequence, many GPs rely solely on, often inaccurate, unstructured clinical assessment [7,8,18,20]. However, diagnosing HF on clinical grounds alone can be unreliable due to difficulty in interpreting signs [21] and differences between doctors in obtaining symptoms and signs [13,22]. Many GPs order a chest x-ray, or arrange an ECG [7]. However, although a normal ECG will exclude LVSD in most cases, changes may be subtle and lack of GP interpretation skills may still require referral for specialist opinion. A normal chest x-ray does not exclude HF [23]. A key dilemma facing GPs is deciding which patients to refer for Echo and when; and lack of a systematic method for guiding the diagnosis of HF presents a further obstacle [7], adding to cost and delay. Diagnostic uncertainty or inaccurate diagnosis can result in diagnosis being delayed until HF symptoms are more obvious and therefore more severe, multiple GP consultations and hospital admissions, or people are treated incorrectly.

A growing body of evidence suggests the potential utility of B-type natriuretic peptides (NPs), namely BNP or NT-proBNP, both released from myocardium in response to wall stretch, as diagnostic cardiac biomarkers of HF. These NP tests provide an exciting opportunity to support the clinical assessment of symptomatic primary care patients, as normal levels can rule out HF given the high sensitivity of these tests (98%) [24], but confirmatory Echo is needed in patients with elevated peptides to confirm the diagnosis [24-28].

There is uncertainty about the best cut-off levels of NPs in primary care and the cost-effectiveness/benefit has not been established. NP testing is under-used because reliable data on BNP and NT-proBNP performance in the diagnosis of HF are limited mainly to epidemiological sub-studies or to prospective validation in emergency department settings [27,29-31], with limited data on test performance within symptomatic patients routinely presenting in primary care [24,28,32]. Best assay cut-offs have therefore been largely imputed and assay performance against or with ECG and symptom score unclear. Moreover, obesity and certain HF medications can lower peptide levels and elevated levels can be associated with unrelated conditions and other factors such as increased age, gender and renal insufficiency [25]. These factors therefore impair the utility of NPs as a diagnostic marker of HF if used alone. The addition of a B-type NP test to the current diagnostic pathway, with specialist referral if test results are abnormal, is a suggested alternative approach that may be superior and cost-effective [33]. However, the cost-effectiveness of NPs versus standard diagnostic triage is not established.

Current consensus suggests a superior approach would be to combine NP testing with standard clinical assessment. In a recent prospective, randomised controlled trial of 305 elderly patients with symptoms of recent onset breathlessness or oedema GP diagnoses were more accurate with NT-proBNP test results in addition to routine clinical assessment than without, mainly due to the ability to correctly rule out HF [28]. A recent meta-analysis concluded that the use of NPs could help reduce the demand for Echo and cardiology referrals [34]. However, determining the optimal manner in which to combine clinical features from clinical assessment and diagnostic tests, including NP tests, remains extraordinarily challenging.

Clinical decision rules (CDRs) are evidence-based clinical tools designed to be used to help clinician decision-making in a standardised and cost-effective manner, and are developed according to strict methodological procedure [35,36]. These clinical tools are based on a parsimonious set of variables that can quantify the contribution from history, physical examination and diagnostic tests. They are developed and evaluated in three distinct stages prior to implementation into a clinical setting: 1) creation of the rule, establishing the independent and combined effect of explanatory variables such as symptoms, signs or diagnostic tests; 2) validation of the rule, establishing the accuracy and reliability of the tool in a separate population; and 3) impact analysis of the rule, establishing impact of applying the rule on patient outcome or health professional behaviour.

A number of CDRs have been developed to diagnose HF, using combinations of signs, symptoms and tests [37-39]. However, a major problem with all the studies is spectrum and referral bias since most were based on observational screening studies rather than symptomatic presenting patients and some were hospital rather than community based. Additionally, the tools are impractical outside a research or emergency department setting as they are based on a substantial number of variables; others rely on clinical signs where there is considerable inter-observer variation, even amongst specialists; and others rely on chest x-ray parameters, which would be difficult to apply in general practice.

Our recent NIHR HTA funded systematic review and independent patient data and meta-analysis [40,41] addressed this issue. We found individual symptoms (such as breathlessness and fluid retention) and signs (such as resting tachycardia and raised jugular venous pressure) are generally weak predictors of HF. Both ECG and BNP have high sensitivity for HF and are good tests at ruling out the diagnosis but BNP is more accurate than ECG. We found BNP and NT-proBNP to be of similar accuracy.

Our systematic review [40] identified one unpublished study which had developed a decision tool based on simple clinical features [42]. In our individual patient data analysis [40,41] we further developed this tool and validated it on other primary care data sets. We found that a simplified model, based upon simple clinical features (Male gender, history of myocardial Infarction, basal Crepitations, oEdema: ‘MICE’) and BNP derived from one data set, was found to have good validity when applied to other data sets, with the area under the curve between 0.84 and 0.96, and reasonable calibration. A model substituting ECG for BNP was less predictive. Our systematic review concluded that BNP could substitute for ECG for determining referral to Echo and some patients could be referred with no prior tests on the basis of clinical features alone.

We shall establish the clinical utility of B-type NP tests in informing the diagnosis of diastolic HF as well as LVSD and valve disease. Additionally, we shall determine the probability thresholds of the CDR above which Echo would be the most cost-effective diagnostic strategy, taking into account patient quality of life and survival. The results will contribute to scientific progress by solving the problem wherein GPs have clinical uncertainty about whether an Echo should be done or not for a patient whom they suspect may have HF. There is now an opportunity to provide these data and to potentially demonstrate that the CDR can improve patient management concerning diagnostic accuracy, clinical decision-making and cost-effectiveness.

Our study will build upon the current evidence and address the weaknesses in previous work. We have validated the CDR on primary care data sets but further validation in a symptomatic population in the real-life clinical setting is now indicated. Further exploration of the optimal NP cut-offs and further modelling of cost-effectiveness is also needed. We aim to prospectively validate the CDR in this study but GPs will not apply the CDR (applying the rule would be appropriate in an implementation study); GPs will refer all patients suspected of having HF and not previously diagnosed with Echo and we shall collect data on how well the CDR predicts the diagnosis of HF. The CDR’s impact potential will be demonstrated by evaluating whether its sensitivity and specificity is superior to that of GPs’ (unaided) decisions. Given the risk of delayed diagnosis of HF, GPs do not have clear guidance on whom to refer for further evaluation. Improving the ability of GPs to appropriately identify patients suspected of having HF is crucial not only to avoid unnecessary hospital admissions and reduce patient burden, but also to improve the quality of care for patients presenting to primary care with suspected HF.

We propose the following objectives:

1. To prospectively validate the performance of the CDR and compare it to using a natriuretic peptide assay alone on the diagnostic accuracy of HF in primary care

2. To determine if the CDR, or natriuretic peptide assay can be used in routine clinical practice to establish referral on for echocardiography in patients presenting with symptoms suggestive of HF

3. To quantify the most reliable cut-off levels of the natriuretic peptide assay in a group of symptomatic presenting patients

4. To model the cost-effectiveness of using the CDR in primary care

## Methods/design

### Study design and setting

REFER is a prospective, observational, diagnostic validation study of a CDR, natriuretic peptide or their combination, for diagnosing heart failure in primary care. The study will be conducted in 30 urban and rural primary care practices in Birmingham, West Midlands, England.

### Study population

All adult primary care patients aged 55 years or over presenting to their GP with recent new onset symptoms of breathlessness, lethargy or ankle oedema of over 48 hours duration, with no obvious recurrent, acute or self-limiting cause will be enrolled.

### Patient eligibility

Patient eligibility will be determined by the GP using information from clinical history and examination and recording clinical judgment via a GP web-based database. The eligibility criteria will be confirmed by investigation and specialist interpretation of clinical assessments. Objective evidence of HF (reference standard) will be determined by a specialist panel that will validate previous diagnosis and investigations.

### Inclusion criteria

• All patients 55 years of age or over presenting to their GP with new onset symptoms of breathlessness, lethargy, or ankle oedema of over 48 hours duration, with no obvious recurrent, acute or self-limiting cause

• Able to give informed consent

### Exclusion criteria

Patients will be excluded if any of the following criteria are met:

• Known pre-existing HF or LVSD of any cause. However, patients with a pre-existing label of HF but without objective evidence (i.e. echocardiography) of this will not be excluded

• Severe symptoms requiring urgent assessment or stabilisation (e.g. breathless at rest, hypotension, confusion)

• Obvious clinically determined alternative diagnoses such as chest infection, exacerbation of chronic obstructive pulmonary disease or asthma

• Recent acute coronary syndrome (within 60 days)

• Major co-morbidity or other alternative diagnoses of no obvious acute and self-limiting cause (e.g. malignancy, severe respiratory disease, renal dialysis, mental health problem)

• Unable to provide informed consent

### Patient recruitment

**Figure 1:**
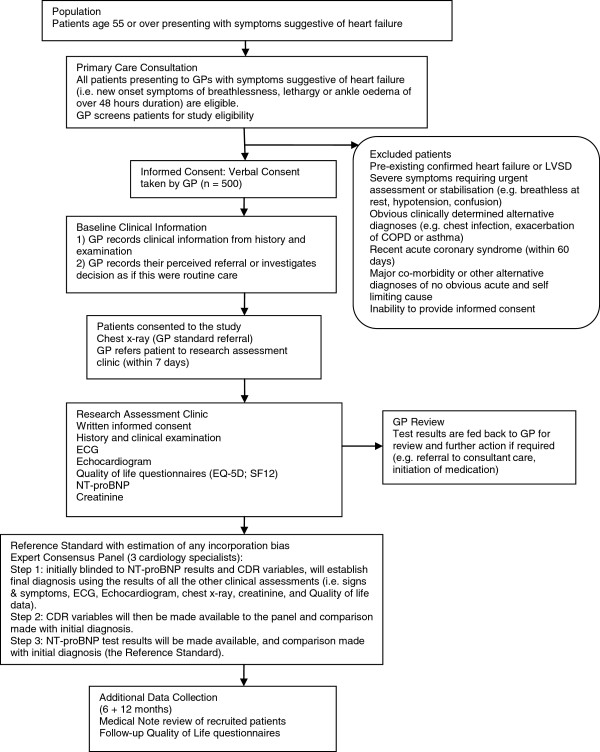
Flow of patient recruitment and data collection.

GPs will give patients a written study information sheet that outlines the nature of the study, including possible benefits and risks, ethics approval, and advice that they can decline to participate or withdraw at any time without this affecting their medical care. GPs will then obtain verbal consent; full informed written consent will be taken subsequently by a research nurse at one of our two research assessment clinics. The flow of patients through the study is shown in Figure 1.

### Referral of patients to research assessment clinics

GPs will refer all eligible patients who have given verbal consent to participate in the study to one of our two research assessment clinics in two ways by: 1) asking the patient to telephone our research team administrator to arrange an appointment within seven days of the initial GP consultation, or the GP obtains patient contact details for entry onto the GP electronic database and the study team telephones the patient; and 2) GP completion of eligibility criteria onto the web-based Case Report Form will act as a referral letter, which we shall check to confirm eligibility and to ensure that the patient has contacted the research assessment team. If a patient changes their mind between agreeing to participate at the GP consultation and before attending their appointment at the research clinic, they are advised that they can cancel by telephoning either the research team or their GP. The patient can then re-consult with their GP, if necessary. Patients who decline to take part in the study will be managed as usual practice by GPs. When eligible patients decline to participate in the study or patients do not meet entry criteria for the study, GPs will complete a weekly electronic notification form of these details. These data will be used to assess potential selection bias.

### Recruitment rate

A total of 500 patients will be recruited, 25 per practice. To allow for delays, the target recruitment rate will be three patients per practice per month, with expected recruitment completed within 18 months. We shall monitor recruitment rates and if we fail to achieve the target accrual rate, additional practices will be invited to participate.

### Clinical decision rule

The CDR, developed from our HTA individual patient data and meta-analysis [40], intended to be used at the start of the diagnostic pathway in primary care [43], states:

Refer straight for echocardiography if the patient has any one of:

• A history of Myocardial Infarction

• Basal crepitations

• Ankle oedema in a male

Otherwise, carry out a BNP (or NT-proBNP) test and refer straight for echocardiography if BNP/NT-proBNP level is above one of three cut-offs set by gender/symptoms recorded in the clinical rule:

• Female without ankle oedema, refer if BNP > 210-360 pg/ml depending upon local availability of echocardiography (or NT-proBNP > 620-1060 pg/ml), or

• Male without ankle oedema, refer if BNP > 130-220 pg/ml (or NT-proBNP > 390-660 pg/ml), or

• Female with ankle oedema, refer if BNP > 100-180 pg/ml (or NT-proBNP > 190-520 pg/ml).

### Primary and secondary endpoints

Primary outcomes include:

• Test performance of the CDR, estimating the sensitivity and specificity, positive predictive value and negative predictive value of the CDR for diagnosis of HF in symptomatic patients presenting with shortness of breath, lethargy, or ankle oedema of over 48 hours duration

• Test performance of the diagnostic accuracy of NT-proBNP for diagnosis of HF in symptomatic patients, including sensitivity, specificity, positive predictive value and negative predictive value

• Proportion of patients with LVSD or not (ejection fraction <40%) and HF or not

Secondary outcomes include:

• Combination of the CDR and NT-proBNP

• Modelling of CDR test performance and epidemiological data to ascertain the most cost effective strategy in the diagnosis of HF in primary care, incorporating data on quality of life (EQ-5D and SF12 questionnaires), clinical events and health care resource use

• Reliability of GP clinical judgment alone in diagnosing HF

• Reliability of individual clinical features

• Reliability of ECG interpretation

• Estimation of the best performing cut-offs for NT-proBNP to maximise diagnostic yield and for maximizing cost-effective referrals

• Determine the use of variable echocardiographic markers of diastolic function in the diagnosis of HF with preserved ejection fraction

### Clinical judgment

During the initial consultation, GPs will have identified a patient as eligible for referral to one of the two research assessment clinics (i.e. recent new onset shortness of breath, lethargy or ankle oedema of over 48 hours duration). They will then complete the two clinical judgment sections of the online web-based Case Report Form: 1) details of symptoms, history and patient information, including the predictive clinical features of the CDR; 2) whether they would have made a clinical diagnosis of HF or not and what they would have done routinely with this patient (i.e. investigate, initiate referral, treat, follow-up). Following diagnostic assessment at the research assessment clinic, the NP results will be fed back to GPs and based on those results, GPs will be asked what they would do (refer or not refer to Echo) and if they would amend their original diagnosis.

### Diagnostic assessment

The GP will have arranged for all patients to receive a chest x-ray when verbally consenting patients for referral (as is usual practice). Within seven days of referral, the research assessment clinic team will obtain written informed consent, collect baseline demographics, administer quality of life questionnaires (EQ-5D [44] and SF12 questionnaires [45]), clinically assess patients, perform an ECG and echocardiogram, and take blood for NT-proBNP, along with creatinine for a renal dysfunction test, calculating an eGFR (serum profile). These clinical assessments will be made by a research nurse or clinical research fellow trained in these assessments, including phlebotomy, auscultation and chest examination. The heart sounds and chest sounds for each patient will be recorded digitally and a random sample validated by a Senior Cardiologist blinded to the assessment clinic findings. If the research team believes an early decision on management needs to be taken on the basis of the patient’s symptoms or signs at the research assessment clinic, or the results of any of the investigations, an urgent specialist referral will be organised via the patient’s GP. After we have received the GPs’ clinical judgment in the Case Report Form on what they would do (refer or not refer to Echo), all test results will be made available to GPs.

### ECG assessment and interpretation

A 12-lead electrocardiogram (ECG) will be performed. ECGs will be analysed with diagnostic software and double reported and interpreted by the Echocardiographic Technician and a blinded consultant cardiologist, blinded to each other’s interpretation, the software interpretation and other data (i.e. symptoms, echocardiography, chest x-ray, NT-proBNP results). Inter-observer variability will be recorded and analysed.

### Echocardiographic assessment

Echocardiography will be performed within seven days of GP referral by a BSE Accredited Echocardiography Technician, using a portable high-quality Vivid i Ultrasound machine. The echocardiographic assessment, with objective assessment of left ventricular dimensions and ejection fraction, measurement by an area-length method, will be extended to include assessment of diastolic dysfunction. Echocardiogram results, together with clinical assessment results, will be used to establish the final diagnosis, as the reference standard.

### Questionnaire measures

The quality of life questionnaires will be self-administered in the research clinic and data entered into an electronic database. Follow-up questionnaires will be mailed to patients at 6 and 12 months to provide data for health economic modelling. We shall register participants with the Office of Population & Census Statistics (OPCS), based on our previous studies (e.g. Echoes-X), and the research team will check patient status (dead/alive) before sending follow-up questionnaires to patients.

#### The EuroQol 5D (EQ-5D) questionnaire

The EQ-5D is a widely used patient-based generic questionnaire for self perceived health assessment [44]. There are five domains, including mobility, self care, main activity (i.e. work), leisure activity, pain and anxiety. It describes health-related quality of life, giving a single index score for each health state measured that can be combined to generate a single index where 1 = perfect health and negative scores represent poorer states of health.

#### SF-12 questionnaire

The SF-12 is a widely used and validated short generic questionnaire for measuring health related QoL [45], and has been validated for measuring QoL of patients with cardiovascular disease [46].

### Blood collection and biomarker assessment

We shall collect a 30ml blood sample by venepuncture into EDTA K2 blood tubes to perform a point of care NT-proBNP test using a Cobas h 232 Reader and Roche Diagnostics CARDIAC proBNP test strips, for immediate results, and to perform a serum creatinine test to exclude renal dysfunction and calculate an eGFR (serum profile). Blood samples will be sent to Midlands Pathology for spinning and storage for batch analysis, and stored at −80°C for future analysis.

### Reference standard for presence/absence of heart failure

An independent expert consensus panel comprising three cardiology specialists will determine the final diagnosis of LVSD or not (ejection fraction <40%) and HF or not, based on internationally accepted definition [47], with differences resolved by consensus. In order to reach an accurate diagnosis the consensus panel need all clinical and test information but this could introduce incorporation bias. To minimise this but provide fuller information for the consensus panel they will receive information in 3 steps. In Step 1, echocardiography results will be provided along with all other clinical information except the NT-proBNP test results and clinical variables included in the CDR, namely, history of myocardial infarction, gender, basal lung crepitations and ankle oedema. The consensus panel will reach a decision on whether or not heart failure is present initially without these data. In Step 2, CDR clinical variables will then be made available to the expert panel and comparison made with the initial assessment. In Step 3, NT-proBNP test results will be provided and the consensus panel asked whether this changes their opinion. The primary reference standard for the study is therefore Step 3 where all clinical and test information is available to the consensus panel. However, we will also be able to accurately estimate any incorporation bias that may have related to this reference standard based upon Steps 1 and 2.

### Definition of heart failure

Clinical HF will be defined using the European Society of Cardiology guidelines: “HF is a syndrome in which the patients should have the following features: symptoms and signs of HF and objective evidence of an abnormality of the structure or function of the heart at rest” [14].

### Medical note review

Medical note review, obtained from GP notes, on recruited patients will be performed at 6 and 12 months. Data on medications, hospital and nursing home admissions, A&E attendance, referrals presentation with new symptoms/complications and death will be recorded. We shall use these data (i.e. clinical events and resource use) in the economic modelling of outcomes associated with the use of the CDR.

### Sample size

Thirty urban and rural general practices in the West Midlands will be asked to participate to recruit 500 symptomatic patients. A search of routine practice morbidity data suggest that in a practice of 6,000 patients, around 60 patients over age 55 per year will present with new onset breathlessness. Breathlessness is the commonest of the three most likely symptoms of heart failure (others are lethargy or ankle swelling) and therefore these estimates on the rate that symptoms present will be the minimum rates. Assuming a 60% response rate then it would take at least nine months to recruit at least 25 such patients per practice. All practices will stop active patient recruitment at the end of 18 months. Calculations are based on sensitivity of 94% and specificity of 48% obtained from application of the CDR in our HTA funded individual patient data meta-analysis [40] and the prevalence of heart failure in a symptomatic population of 30%. A sample size of 500 patients with HF symptoms will therefore be sufficient to estimate the sensitivity of the CDR to within 4% and specificity to within 6% at the 95% confidence level.

### Statistical analysis

Data will be analysed using SAS and STATA software. Patients with symptoms of HF that are referred to Echo via the CDR will be classed as Test disease present and the remaining patients classed as Test disease absent. The Observed disease present or absent will be determined by the expert panel following Echo and other clinical assessments. Crosstabulation of Test versus Observed disease status will enable calculation of sensitivity (true positive rate), specificity (true negative rate), positive predictive value (PPV: proportion with a positive test result who actually have the target condition), negative predictive value (NPV: proportion with a negative test result who do not have the target condition), and likelihood ratios for testing the performance of the CDR. 95% confidence intervals for these performance statistics will be calculated using the binomial exact method.

To confirm whether the NT-proBNP cut-offs in the CDR are optimal in the real life clinical setting, an additional ROC curve analysis of NT-proBNP to predict HF will be performed. Analysis will compare the CDR performance against the step 1 reference test alone; against the step 1 reference test plus clinical features of the CDR (step 2); and against the step 1 reference test plus the CDR and the NT-proBNP result (the reference standard, step 3). Step 3 is the primary reference standard for analysis. This will allow us to: 1) quantify the effects of any incorporation bias; 2) explore the impact that availability of NT-proBNP test result would have on the reference standard diagnosis of HF. Comparison of the GPs’ and researcher’s clinical findings (lung crepitations, ankle oedema, decision to refer to Echo) will be assessed by the kappa statistic. Logistic regression will be used to identify which diastolic parameters of echocardiography are independently associated with the diagnosis of heart failure with preserved ejection fraction.

### Health economic analysis

A decision tree will be used to assess the cost-effectiveness of the CDR [48]. The prevalence of heart failure in patients presenting to primary care will be determined both from the study cohort and from a review of the epidemiological literature. The probability that patients with and without heart failure will be referred for echocardiography will be determined based on the test characteristics of both the CDR and of existing practice. The decision tree may be further refined depending on the power of the available data from the study; for example, distinguishing between patients with HF of different levels of severity (such as LVSD and HFpEF patients).

Cost and quality of life implications for patients at different branches of the decision tree will be extrapolated based both on data collected during the study and from the literature. Prospectively collected data on quality of life, clinical events and health care resource use will be used to estimate outcomes associated with using the CDR, NT-proBNP, their combination, or continuing with current practice. Since the study does not capture the full details of every acute event in the cohort, the cost and quality of life implications of such events will be imputed from the literature and standard UK sources of health economic information [49,50].

Outcomes associated with current practice will be estimated by using GP reported clinical judgment to predict their intentions for patients in the absence of using the CDR. In addition, the model will allow exploration of the effect on cost effectiveness of hypothetical scenarios involving altering the threshold peptide value for referral to echocardiography. Decreasing the threshold will cause more people to be referred for echocardiography, hence increasing costs but also improving outcomes. By using a suitable threshold cost per QALY cut-off (such as the threshold of £20,000 - £30,000 used by the National Institute of Health and Clinical Excellence) [51], the optimal threshold peptide value for referral can be estimated [52].

Costs will be evaluated from a health care provider perspective, with a lifetime time horizon. The effect of uncertainty in parameter values will be quantified by both univariate and probabilistic sensitivity analysis, and will be summarised using appropriate methods (cost-effectiveness plots and/or cost-effectiveness acceptability curves) [48].

### Ethical considerations

The study has full approval from South Birmingham Research Ethics Committee, reference number 09/H1207/121. We shall ensure that participation in the study does not lead to inferior medical care by performing the diagnostic assessments within seven days of GP referral and by informing the patient’s GP of the clinical assessment results and any clinical abnormalities uncovered within seven days. Therefore, study patients will receive a higher standard of care than would be likely in routine practice. Post-study medical care will be provided by patients’ GPs.

### Participant consent

We shall ensure that patients are fully informed about all aspects of the study prior to obtaining informed consent. Study duration, assessments and the voluntary nature of participating will be discussed before obtaining written informed consent. We shall record all requests to withdraw from the study.

### Potential risks and burdens

There should be no risk to patients since a full diagnostic assessment, including the reference standard diagnosis, which is non-invasive, is carried out on all patients in the study and in a timely manner. Therefore, patients are receiving a higher standard of care than would be likely in routine practice.

### Data quality assurance

The data manager will oversee all data collection with built in validation and regular reports to ensure the integrity of data. The database will be monitored exclusively by the data manager and the statistician. Data will be extracted from the database and imported into statistical software packages, SAS and STATA. The statistician will then perform further validation checks through exploratory data analysis, identifying any blank fields, outliers and logical inconsistencies between fields. Any problems detected will be then verified and corrected by the data manager. The project manager will ensure that the conduct of the study complies with the currently approved protocol, with Good Clinical Practice, and all applicable R&D regulatory procedures. All study activities will be performed in accordance with the University of Birmingham Standard Operating Procedures.

### Research governance

The study will be conducted in accordance with the Guidelines for Good Clinical Practice, the Research Governance Framework for Health and Social Care, 2005 and the Data Protection Act 1998. Patient involvement has ensured that both the patient information and consent forms are relevant to the patients the study attempts to improve services for. All relevant regulatory approvals have been sought prior to commencing the study and the study registered with the UK Clinical Research Network (UKCRN No: 7944). We have obtained ISRCTN registration (ISRCTN17635379) prior to patient enrolment.

The study will be conducted in compliance with Data Protection legislation, with particular attention given to the emphasis on privacy and on processing of personal data extending to disposal or destruction and disclosure to a third party. Participants shall be informed that information may be accessed during the study by Regulatory Authorities or the relevant NHS Trust in compliance with the Data Protection legislation. Data processing and linkage of personal information will be subject to the strictest ethical safeguards of anonymity, with documents assigned a numerical code. All data shall be held securely and treated with the strictest confidentiality.

### Indemnity and sponsorship

The University of Oxford has arrangements in place to provide for negligent and non-negligent harm arising from participation in the study for which the University is the Research Sponsor. NHS indemnity operates in respect of the clinical treatment which is provided.

### Role of the funding source

The funders have no involvement in the following activities: study design; collection, analysis or interpretation of data; report writing or submission of manuscripts for publication.

## Discussion

The REFER Study is a multicentre, Phase II study designed to prospectively validate a CDR to improve the diagnosis of heart failure in primary care. HF is a diagnostic dilemma for the general practitioner [6,8,13,53]. In the absence of an accurate method of identifying patients with HF, triage of patients for echocardiography in primary care is variable. As a result HF diagnosis is often delayed, misdiagnosed, or treated incorrectly [53]. The main characteristic of the REFER study is that it examines a symptomatic adult population presenting in general practice.

A major problem with all the studies we uncovered in our review of the diagnostic test systematic reviews is spectrum and referral bias since most were based on observational screening studies rather than symptomatic presenting patients, and some were hospital rather than community based [40]. Only a prospective study design with consecutively recruited symptomatic patients can avoid or minimise these potential biases. Another major strength of the REFER study is that the study has been designed according to strict methodological standards to determine the diagnostic accuracy of the CDR [35,43,54-56]. Moreover, the study’s design will provide data on the impact of the availability of the NT-proBNP result on the reference standard diagnosis of heart failure. These data are of clinical significance, given that the current NICE algorithm for heart failure diagnosis envisages NT-proBNP being used principally as a triage test to determine which patients should have echocardiography [16]. However, in diagnosis of heart failure with preserved ejection fraction, it may be that a raised natriuretic peptide level is of diagnostic value in its own right. Further, the results will provide data on optimum natriuretic peptide cut-offs which, as far as we are aware, are not yet available. Additionally, the planned modelling will indicate whether further research is not necessary if the strategy dominates or the technology fails. An intermediate result would enable accurate powering for any future Phase III trial.

### Limitations

This study has potential limitations that apply to validating CDRs. Reference standard misclassification is an inherent problem of diagnostic accuracy studies that may introduce bias [57,58]. The prospective study design will allow us to ensure that all symptomatic patients presenting with new onset symptoms suggestive of heart failure will receive identical standardised assessments.

This will ensure that all patients are diagnosed in a consistent manner in order to avoid introducing variation into the reference standard. Our reference standard also incorporates multiple assessments, thereby minimising potential reference standard misclassification [59]. Another possible limitation concerns incorporation bias. This occurs when knowledge of the result of the index test influences the reference standard test. The CDR incorporates variables from the history, examination and tests that would normally be included in a reference standard. To minimise the potential for bias our expert consensus panel will initially receive the clinical information and investigation results about the patient with the exception of the information that comprises the CDR (namely history of myocardial infarction; gender; basal lung crepitations and ankle oedema) and the NT-proBNP result. They will be asked to reach a decision on whether or not HF is present initially without these data. They will then be given the clinical features of the CDR score and asked if this influences their decision, and then the results of the NT-proBNP test. This stepped reference standard will allow us to quantify the effects of any incorporation bias.

Selection bias is a key issue in diagnostic accuracy studies. This bias would occur if GPs enrolled only those patients they believed to have a high probability of heart failure. This would mean more symptomatic or more severe cases would dominate the study sample and result in an over-estimation of the test-accuracy of the CDR [57,58]. In our study, the consecutive inclusion of all symptomatic patients will minimise the risk of introducing this type of bias. GPs will be asked to refer all patients over 55 years of age presenting with the target symptoms regardless of whether or not heart failure is suspected by the doctor and excluding patients with pre-existing confirmed heart failure. Verification bias is also a potential limitation and may occur if patients with low pre-test probability of heart failure were to be excluded from undergoing reference standard diagnostic testing to verify the diagnosis of heart failure. This would result in an over-estimation of test sensitivity [58]. Performing all diagnostic assessments on all patients included in the study will minimise this potential source of bias.

## Conclusions

We aim to prospectively validate our previously derived (Phase I) novel, simple CDR in a new set of symptomatic patients in clinical practice. If shown to be cost-effective, the CDR will support GPs in prioritising which patients with symptoms suggestive of HF should be referred on for echocardiography. The CDR has the potential to improve patient care and GP clinical decision-making, reduce diagnostic uncertainty and variability in practice, increase the speed with which people with HF commence treatment, and lower NHS costs.

### Publication policy and dissemination

Recommended practice in journal guidelines will be followed in relation to authorship. We shall comply with authorship guidelines suggested by the International Committee of Medical Journal Editors, available at http://www.icmje.org.

## Competing interests

RH and MRC have received research funding in the form of reduced cost or free natriuretic peptide assays from Roche Diagnostics. Both have also received occasional speaker fees and symposia expenses from Roche and Bayer Diagnostics.

## Authors’ contributions

RH, LT, MC and JM conceived of and designed the study. LT, AKR, JM, MRC, JD, PMB, CT and FDRH were grant applicants. LT was responsible for drafting the protocol and manuscript. AR (Statistician) is responsible for the statistical analysis plan. PB (Health Economics) is responsible for the economic modelling plan. All authors read and approved the final manuscript.

## Pre-publication history

The pre-publication history for this paper can be accessed here:

http://www.biomedcentral.com/1471-2261/12/97/prepub
